# The Incidence of Impacted Maxillary Canines in a Kosovar Population

**DOI:** 10.1155/2014/370531

**Published:** 2014-07-08

**Authors:** Ali Gashi, Blerim Kamberi, Resmije Ademi-Abdyli, Ferijale Perjuci, Arjeta Sahatçiu-Gashi

**Affiliations:** ^1^Department of Oral Surgery, University Dentistry Clinical Center of Kosovo, Prishtina, Kosovo; ^2^Department of Conservative Dentistry & Endodontics, University Dentistry Clinical Center of Kosovo, Rrethi i Spitalit p.n., 10000 Prishtina, Kosovo; ^3^Department of Orthodontics, University Dentistry Clinical Center of Kosovo, Kosovo

## Abstract

*Aim*. The purpose of this study was to investigate the incidence of impacted maxillary canines in a Kosovar population. *Materials and Methods*. The study consisted of a retrospective analysis of the records of 8101 patients treated in the University Dentistry Clinical Center of Kosovo between August 2001 and February 2004. The chi-squared test was used to examine potential differences in the distribution of impacted maxillary canines stratified by gender, age, location (left or right), and position. *P* < 0.05 was accepted as statistically significant. *Results*. It was found that the incidence of impacted maxillary canines was 1.62%. Of the 131 impacted maxillary canines, 101 were in female patients and 30 were in male patients, a statistically significant difference. Ages were in the range of 9 to >20 years, with a mean age of 24.38 ± 8.09 years. Of these subjects, 99 (75.57%) had unilaterally impacted maxillary canines, while 32 (24.43%) had bilateral impactions, a statistically significant difference (*P* < 0.00002). Impacted canines in 92 subjects (70.2%) were palatally placed, and 18 (13.7%) were labially placed. This difference was also statistically significant (*P* < 0.00001). *Conclusion*. The incidence of impacted maxillary canines in the sample Kosovar population was 1.62%, which is comparable to the findings from previous studies.

## 1. Introduction

Impaction of canine teeth is a well-documented phenomenon, particularly in the recent literature. The occurrence of maxillary canine impaction is considerable and its frequency increases with other genetically associated dental anomalies.

The etiology of impacted maxillary canines is still unknown [1, 2]. Possible causes may include one or more of the following local factors and systemic conditions: inadequate space for eruption or early loss of primary canines; abnormal position of the tooth bud; the presence of an alveolar cleft, a cystic lesion, or neoplasm; ankylosis; dilacerations of the root; an iatrogenic or idiopathic cause; endocrine deficiencies; malnutrition; fever; or irradiation [2–5]. Peck et al. [6] suggested that palatal canine impaction is genetic in origin, whereas labial impaction is due to inadequate arch space.

Numerous studies have been conducted to investigate the incidence and prevalence of impacted maxillary canines in different populations [7–11]. Impacted canines are more commonly seen in female than in male patients, and there is wide variation among different racial populations [12, 13]. Both the maxillary and mandibular canines may be impacted, although maxillary canine impaction is considerably more common [3, 4, 14]. Unilateral impaction is more prevalent than bilateral impaction [2–4], and impaction is ~50 times more frequent in the palate than in the buccal vestibule [3, 4, 10].

However, despite information from other ethnic groups, studies on impacted maxillary canines have not yet been performed in Kosovo. The purpose of this study was to determine the incidence of impacted maxillary canines in a Kosovar population.

## 2. Material and Methods

This study was conducted on a Kosovar population that had been treated in the Oral Surgery Department at the University Dentistry Clinical Center of Kosovo from August 2001 to February 2004. The study consisted of a retrospective analysis of the records of 8101 patients. These records were examined to reveal any evidence of impacted maxillary canines (i.e., visual inspection, palpation, and/or radiographs). Clinical examination was done by conventional methods and included whole-arch inspection; palpation to identify any retained deciduous canine; visualization of the canine “bulge,” splaying of the lateral incisors, lost space, crowding, or fibrous tissue overlying the canine region, and mobility of the primary canines; and a review of the patient's chronological age and history of dental eruption/exfoliation patterns. According to Ericson and Kurol [15], clinical examination should be supplemented with a radiographic evaluation to produce an accurate diagnosis. Panoramic radiographs were taken using a Planmeca 2002 CC Proline (Helsinki, Finland) using Kodak dental films (T-MATE; Kodak, New York, USA). Other radiographs, including anterior occlusal radiographs, were used to determine the position of the impacted canine by parallaxing. These were acquired using a Philips X-ray machine (Philips Medical Systems, London, UK) with Kodak dental films (Kodac E-speed Plus). All films were processed in a XR-24 Nova machine (Durr Dental, Bietigheim, Germany) using Durr dental developer and fixer. All radiographs were placed on a viewing screen and the area surrounding the radiographs was shielded with a dark material to block interfering lateral light and improve viewing contrast. All radiographs were assessed by two experienced oral surgeons.

Data were processed in a Microsoft Excel 2007 worksheet. The chi-squared test was used to reveal any differences in the distribution of impacted maxillary canines when stratified by gender, age, location (left or right), and position. A *P* value of <0.05 was accepted as statistically significant.

## 3. Results

From the 8101 patients, 131 (1.62%) were found to have impacted maxillary canines. Of these, 101 were female and 30 were male, which was a statistically significant difference (*P* < 0.0001) (Table 1). Ages were in the range of 9 to >20 years, with a mean age of 24.38 ± 8.09 years (Table 2). In 99 patients (75.57%), we found unilateral impaction, whereas the remaining 32 (24.43%) were bilateral (Table 3). This difference was also statistically significant (*P* < 0.0001). Among the 99 unilaterally impacted canines, 57 were on the left side and 42 were on the right side.

**Figure 1:**
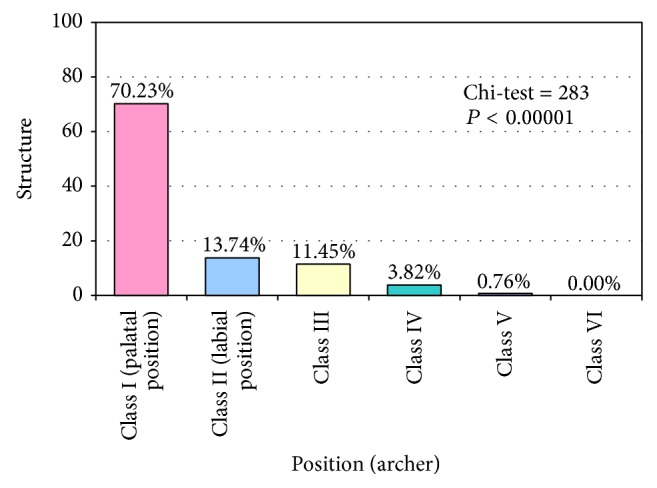
Distribution of impacted maxillary canines according to position in the arch.

**Figure 2:**
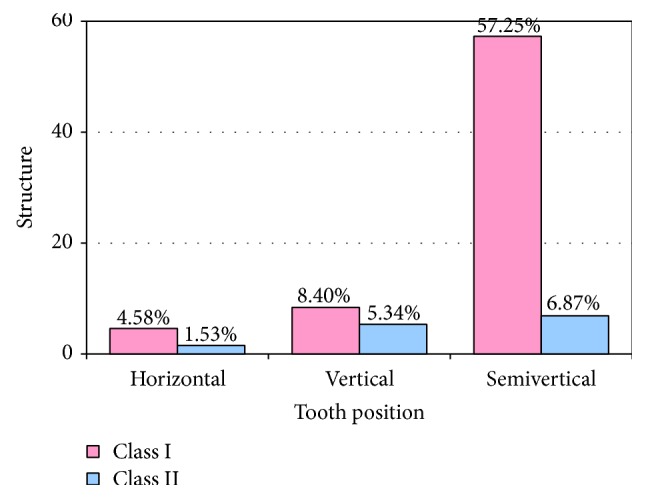
Distribution of impacted maxillary canines according to position in the arch (classes I and II).

In 92 cases (70.2% of total), the impacted tooth was palatally positioned (class I, according to the classification of Archer [16]). Seventy-five of these were in a semivertical position, whereas 11 and 6 were in vertical and horizontal positions, respectively. A further 18 canines (13.7% of total) were labially positioned (class II), with nine being semivertical and seven being in a vertical position (Figures 1 and 2). There were 15 cases (11.5% of total) in which the crown of the impacted canine was palatally positioned but the root extended between the adjacent teeth to end labially (class III). Five canines (3.8%) were impacted within the alveolar process in a vertical position between the incisors and first premolars (class IV). Finally, there was a single case of an ectopic canine (class V) (Figure 1). The chi-squared test revealed that the proportion of impacted canines in each position was statistically significantly different (chi-test = 283, *P* < 0.00001).

## 4. Discussion

Comparison of the results from this study with those reported previously is complex because of differences in sample size, grouping methods, clinical examination methods, and the radiographic techniques used to make the diagnosis.

However, numerous studies have been conducted to investigate the incidence and prevalence of impacted maxillary canines in different populations. The prevalence of maxillary canine impaction appears to vary within a range of 0.9–3.5%, depending on the population examined [7, 8, 11, 14, 17]. In our study, the overall incidence of impacted maxillary canines was 1.6%, suggesting that ethnicity and geographic location have little influence on the incidence of maxillary canine impaction. Female patients have been reported to be more commonly affected [17–21], and our results support this conclusion, with a female : male ratio of 3 : 1. It is possible that this higher frequency in female patients is associated with the smaller cranium in female patients, which may lead to diminution of the facial skeleton [12] and the jaws. This would be expected to increase the probability of maxillary canine impaction. Other authors have hypothesized that the higher female incidence may simply reflect a trend whereby female patients are more likely to seek orthodontic treatment and thus have their impacted canines discovered [7, 22].

Most of the studies published on impacted maxillary canines have dealt with characteristics of unilateral impactions [17, 18, 23], although others conclude that bilateral impaction is more usual [10]. Our findings are in line with previous results suggesting that unilateral impaction is more prevalent than bilateral. Furthermore, the position of the impacted maxillary canines varied greatly. In a European population, palatal canine impaction was around five times more frequent than in an Asian population [6]. In contrast, Kim et al. [24] argue that there is a threefold greater tendency for labial impaction in a Korean population. These differences likely relate, at least in part, to racial differences in jaw bone structure. The report by Zhong et al. [25] strongly supports this opinion, finding that the Chinese also exhibit a greater prevalence of labial impactions (2.1 times more than palatal). In the present study, 70.23% of canines were palatally impacted, with 13.74% impacted labially. This finding is comparable to previous reports evaluating the positional distribution of impacted maxillary canines [19, 26].

The prevalence of the impacted maxillary canine has been investigated extensively elsewhere, but never in a Kosovar population. The present study found a relatively high frequency of impacted maxillary canines in a Kosovar population. However, the study has several limitations, including difficulty in comprehensively tracing every appropriate dental record, note, and orthopantomographs. Some dental records also contained incomplete data. Further studies are likely to be required to identify the etiology behind the high prevalence of impacted maxillary canine teeth in Kosovar subjects.

## 5. Conclusion

This present study concluded that

1. the incidence of impacted maxillary canines was 1.6%;
2. the most affected gender of impacted maxillary canines is the female;
3. the impaction had occurred more unilaterally than in both sides;
4. the most frequent location of impacted canines was palatal.

## Figures and Tables

**Table 1:** Distribution of impacted maxillary canines according to sex.

Sex	*N*	*n* (impact canines)	(%)
Female	4851	101	2.08
Male	3250	30	0.92
Total	**8101**	**131**	**1.62**

Chi-test		15.71	
*P*		<0.0001	

**Table 2:** Distribution of impacted maxillary canines according to age.

Age	*N*	*n* (impact canines)∗	(%)	Mean∗∗	SD
9–14	1233	6	0.48	13.17	0.75
15–20	2134	40	1.87	17.6	1.72
>20	4734	85	1.79	28.36	7.28
Total	**8101**	**131**	**1.62**	**24.38**	**8.09**

^*^Chi-test = 11.74, *P* = 0.0028.

^**^One-way ANOVA, *F* = 4978.04, *P* < 0.0001.

One-way analysis of variance (abbreviated one-way ANOVA) is used to compare means of two or more samples (using the *F* distribution).

**Table 3:** Distribution of impacted maxillary canines according to side.

Side	*N*	(%)
Left	57	43.51
Right	42	32.06
Both sides	32	24.43
Total	**131**	**100**

Chi-test	26.4	
*P*	0.000002	
